# The Biological Activity of *Ganoderma lucidum* on Neurodegenerative Diseases: The Interplay between Different Active Compounds and the Pathological Hallmarks

**DOI:** 10.3390/molecules29112516

**Published:** 2024-05-26

**Authors:** Wenhui Lian, Xu Yang, Qidong Duan, Jie Li, Yuting Zhao, Chunhui Yu, Tianzhu He, Tianxia Sun, Yu Zhao, Weinan Wang

**Affiliations:** 1 Jilin Ginseng Academy, Changchun University of Traditional Chinese Medicine, Changchun 130117, China; lianwenhui1014@126.com (W.L.); yangxu04@126.com (X.Y.); duanqd04@163.com (Q.D.); libaozi2333@126.com (J.L.); 15944557986@163.com (Y.Z.); chunhui_yu02@163.com (C.Y.); nanw2051@gmail.com (T.H.); 2 Guangdong Key Laboratory for Research and Development of Natural Drugs, School of Pharmacy, Guangdong Medical University, Dongguan 523808, China

**Keywords:** *Ganoderma lucidum*, neurodegenerative diseases, bioactive compounds, neuroprotection, therapeutic potential

## Abstract

Neurodegenerative diseases represent a cluster of conditions characterized by the progressive degeneration of the structure and function of the nervous system. Despite significant advancements in understanding these diseases, therapeutic options remain limited. The medicinal mushroom *Ganoderma lucidum* has been recognized for its comprehensive array of bioactive compounds with anti-inflammatory and antioxidative effects, which possess potential neuroprotective properties. This literature review collates and examines the existing research on the bioactivity of active compounds and extracts from *Ganoderma lucidum* in modulating the pathological hallmarks of neurodegenerative diseases. The structural information and preparation processes of specific components, such as individual ganoderic acids and unique fractions of polysaccharides, are presented in detail to facilitate structure–activity relationship research and scale up the investigation of in vivo pharmacology. The mechanisms of these components against neurodegenerative diseases are discussed on multiple levels and elaborately categorized in different patterns. It is clearly presented from the patterns that most polysaccharides of *Ganoderma lucidum* possess neurotrophic effects, while ganoderic acids preferentially target specific pathogenic proteins as well as regulating autophagy. Further clinical trials are necessary to assess the translational potential of these components in the development of novel multi-target drugs for neurodegenerative diseases.

## 1. Introduction

Neurodegenerative diseases (NDDs) refer to a group of neurological disorders that involve the progressive loss of neurons in the central nervous system (CNS) or peripheral nervous system (PNS). These diseases are distinguished by irreversible damage and loss of CNS cells. NDDs display considerable variability between individuals, have a multifaceted etiology, and manifest in a range of clinical symptoms [1]. Recent studies indicate that NDDs arise from a variety of mechanisms, including neuroinflammation, oxidative stress, mitochondrial dysfunction, accumulation of pathogenic proteins, genetic mutations, and deficits in axonal transport, among others [2,3,4,5,6,7]. Despite significant progress in understanding these causative factors, the exact processes leading to neuronal death in NDDs remain elusive. Current medical interventions primarily focus on mitigating the symptoms of NDDs rather than fundamentally altering the underlying neurological conditions of patients. Therefore, there is a growing interest in identifying pleiotropic and advantageous complementary medicines, including natural alternatives, to delay the onset of NDDs [8].

*Ganoderma lucidum* (Curtis) P. Karst., widely known as reishi or Ling Zhi, is a fungal species within the Ganodermataceae family, under the order Polyporales. This species has a global distribution, thriving in both temperate and subtropical regions across North and South America, Europe, and Asia. In ancient China, it had been regarded as an “elixir”, for it can bring longevity to aged people [9]. Nowadays, it is widely applied in nutraceutical products and traditional Chinese medicine, having potential health benefits with antioxidant, antitumor activity, antiviral, antibacterial, anti-inflammatory, and immunomodulatory effects [10]. The chemical composition of *Ganoderma lucidum* is diverse and includes a range of polysaccharides, triterpenoids, peptides, and other phytochemicals. These components, among others, contribute to the pharmacological profile of *Ganoderma lucidum*, influencing various cellular pathways and biological processes [11].

**Figure 1:**
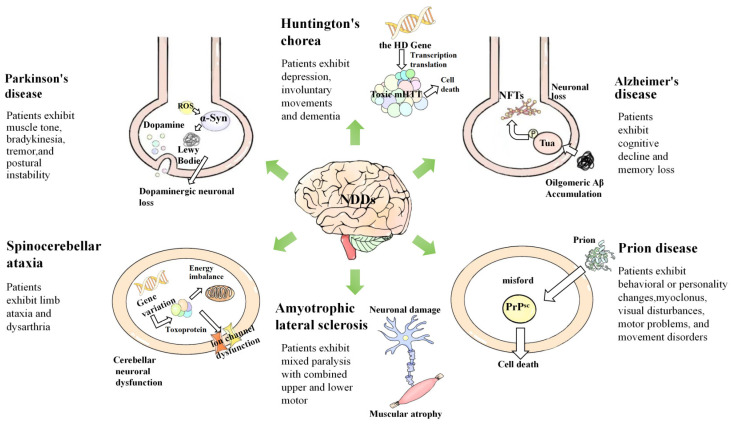
Overview of the main categories of NDDs.

In neurodegenerative diseases, characterized by progressive neuronal loss and dysfunction, the potential of *Ganoderma lucidum’*s active compounds to modulate key pathological hallmarks is of particular interest. These diseases, including Alzheimer’s diseases (AD), Parkinson’s diseases (PD), and Huntington’s diseases (HD) as demonstrated in Figure 1, share common features such as oxidative stress, mitochondrial dysfunction, and abnormal protein aggregation, all of which could be targeted by the mushroom’s bioactive constituents [12]. For instance, a notable study utilizing a Drosophila model of PD provided compelling evidence of *Ganoderma lucidum’*s neuroprotective properties. In this study, it was observed that *Ganoderma lucidum* exhibited a protective effect on dopaminergic neurons, which are crucially affected in PD. The administration of *Ganoderma lucidum* extract (GLE) was found to significantly reduce neuronal damage and loss, highlighting its potential as a therapeutic agent in combating the progression of Parkinson’s disease [13]. Additionally, *Ganoderma lucidum* has shown promising results in mitigating the effects of β-amyloid protein (Aβ) toxicity, a key factor in Alzheimer’s disease pathology. In a study examining stressed neurons, it was observed that *Ganoderma lucidum* effectively reduced the phosphorylation of critical signaling molecules such as c-Jun N-terminal kinase (JNK), c-Jun, and p38 mitogen-activated protein kinase (MAPK). These molecules are known to be involved in the neuronal response to Aβ-induced stress, and their phosphorylation is a crucial step in the pathogenesis of Alzheimer’s disease. The ability of *Ganoderma lucidum* to modulate these signaling pathways suggests a potential therapeutic role in alleviating the cellular stress and neurodegeneration associated with Alzheimer’s disease [14].

Although the effectiveness of *Ganoderma lucidum* against NDDs have been proven on different levels, such as total extracts, bioactive fractions, and single compounds, most of these studies are either shallow bioactivity screening or separately investigate the molecular mechanisms of single compounds without considering how they contribute to the overall efficacy of *Ganoderma lucidum* [15]. Hence in this review, not only was the pathogenesis of certain NDDs investigated, but also the anti-NDD mechanisms of different components in *Ganoderma lucidum* were categorized in patterns of the pathogenic hallmarks. By examining the specific interactions between the components in *Ganoderma lucidum* and these biomarkers, the review aims to shed light on how this traditional medicinal mushroom might offer new strategies for the treatment or mitigation of NDDs.

## 2. Pathogenesis of Neurodegenerative Diseases

**Figure 2:**
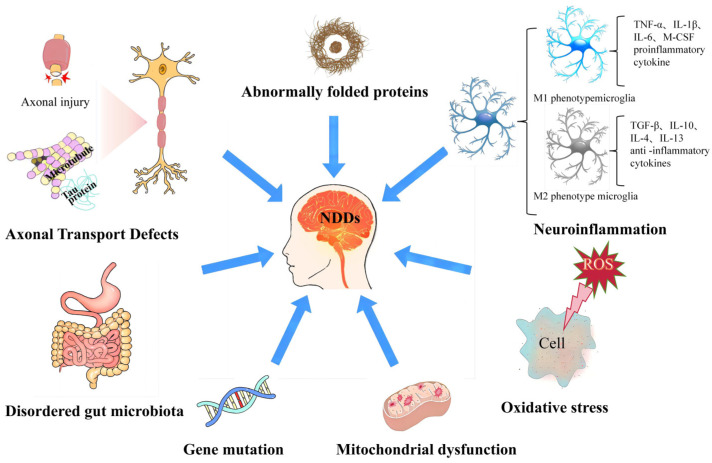
General mechanisms related to NDDs.

Neurodegenerative diseases generally originate from spinal cord and/or brain injury caused by abnormal neuronal death. The likelihood of developing NDDs increases with age, meaning that the population over the age of 65 is considered to be at “risk” for higher rates of AD, PD, and stroke [16]. Patients with NDDs exhibit a variety of pathophysiological symptoms, some of which lead to impaired memory and cognitive skills, while others affect an individual’s behavior, posing a significant threat to public health [17]. The pathological processes of NDDs involve a variety of mechanisms, including neuroinflammation, oxidative stress, mitochondrial dysfunction, abnormally folded proteins, gene mutations, and axonal transport defects, etc. (Figure 2). Different neuroprotective strategies have been established to abrogate these neurotoxic processes, to prevent or delay the disease progression by interfering with one of the specific events involved in this neurotoxic cascade [18].

### 2.1. Neuroinflammation

The inflammation caused by NDDs is a defense mechanism for organisms to resist various harmful stimuli, which can be triggered by a variety of factors such as aging, dementia, trauma, hypertension, depression, diabetes, tumors, infections, and even certain medications [19]. Microglia are the intrinsic neuroimmune cells of the CNS and play an important role in neuroinflammation. Under normal conditions, microglia exhibit low immunoreactivity, but they are easily activated in response to injury or stimulation. Activated microglia can be classified as classical pro-inflammatory M1 or anti-inflammatory M2 types. Microglia of the M1 type exert deleterious effects, while microglia of the M2 type exert neuroprotective and regenerative effects. Prolonged activation of microglia may lead to aberrant phagocytosis processes, which may result in the development of NDDs [20]. Under such conditions, phagocytosis of microglia targets living neurons, neural progenitor cells (NPC) and glioma cells, leading to neuronal loss in the CNS [21]. It has been demonstrated that neuroinflammation and the subsequent neuronal degeneration can be alleviated by regulating the status of microglia [22,23]. Therefore, it is of great importance to investigate drugs targeting such processes in the treatment of NDDs.

### 2.2. Oxidative Stress

Oxidative stress-induced brain damage is a major cause of NDDs [24]. The CNS is susceptible to oxidative stress damage due to the overproduction of reactive oxygen species (ROS) [25]. ROS can lead to protein denaturation, lipid peroxidation, apoptosis and DNA damage [26]. In addition, ROS can directly bind to the mitochondrial membrane, leading to mitochondrial structural and functional disorders. This triggers a cascade of events, including decreased levels of acetylcholine and dopamine, which are directly linked to cognitive dysfunction and neuronal loss, thereby playing a key role in the pathogenesis of several NDDs [27].

While targeting oxidative stress in NDDs holds therapeutic promise, it is beset by several challenges in the setting of drug development. Firstly, the complexity of the oxidative stress pathways presents a significant challenge. The intricate interplay between different ROS and their diverse cellular targets makes it difficult to pinpoint specific molecular interventions. Targeting one aspect of oxidative stress may inadvertently exacerbate another, leading to unintended consequences [28]. Additionally, the heterogeneity of NDDs adds another layer of complexity. Diseases like PD and AD may have common pathological hallmarks, such as oxidative stress, but they also have distinct pathological and molecular features. This necessitates the development of specific drugs tailored to each disease, rather than a one-size-fits-all approach [29]. Furthermore, the dynamic and progressive nature of NDDs means that the window for effective intervention may be narrow. The identification of the stage at which oxidative stress plays a critical role is crucial for the timely application of therapeutic agents. Late-stage intervention might be less effective due to the irreversible damage already inflicted on neuronal structures [30]. These challenges necessitate a multifaceted and nuanced approach to drug development in this field.

### 2.3. Mitochondrial Dysfunction

Mitochondria, as the center of cellular energy production, are essential for maintaining the structure and function of neurons. Damage to mitochondria can lead to chaos in the cellular redox environment and inadequate supply of adenosine triphosphate (ATP). At the same time, dysfunctional mitochondria are recognized by the body’s immune system, which triggers an inflammatory response. Persistent mitochondrial damage can lead to chronic inflammation and neuronal cell death, which may be the leading cause of NDDs in the CNS [30,31].

In addition, mitochondrial autophagy is a process in which cells selectively remove senescent or damaged mitochondria through the autophagolysosome pathway, thereby playing an important role in cell homeostasis. A large number of studies have shown that mitochondrial autophagy is inextricably linked to the occurrence and development of NDDs. The activation of mitochondrial autophagy or improvement in abnormal mitochondrial autophagy can alleviate the neurological damage caused by ill-defined mitochondria [32,33,34,35].

The strategies aiming at alleviating mitochondrial dysfunction in the treatment of NDDs are multifaceted. They involve enhancing mitochondrial biogenesis and function, modulating mitochondrial dynamics, activating mitochondrial autophagy, reducing oxidative stress with targeted antioxidants, and inhibiting the opening of mitochondrial permeability transition pores (mPTPs). These approaches, alone or in combination, hold promise for mitigating the progression of neurodegenerative diseases [36].

### 2.4. Abnormally Folded Proteins

Abnormally folded proteins such as Aβ, phosphorylated tau protein (p-tau), alpha-synuclein protein (α-Syn), and huntingtin protein have been recognized as common inducers of NDDs [37,38].

Aβ is a peptide produced by the cleavage of APP, a large type I transmembrane protein found in neurons. It plays a central role in the pathogenesis of AD. In AD, the 42-residue variant of the Aβ peptide (Aβ_1–42_) can aggregate to form dimers, oligomers, and protofibrils in a process known as amyloidosis. These aggregated forms of Aβ can disrupt cellular processes and induce oxidative stress, inflammation, and neuronal apoptosis, leading to the neurodegeneration observed in AD [39]. Tau is a microtubule-associated protein predominantly found in neurons, where it stabilizes microtubules and plays a key role in maintaining neuronal structure and function. In healthy neurons, tau protein undergoes regulated phosphorylation, which is essential for its normal functioning. However, in pathological conditions, hyperphosphorylation of tau occurs, leading to its aggregation [40]. The abnormally folded tau protein plays a multifaceted role in the pathogenesis of NDDs. Its hyperphosphorylation and subsequent aggregation disrupt microtubule stability, contribute to neuronal dysfunction and cell death, and potentially facilitate the spread of pathology throughout the brain [41]. As part of the pathogenesis of PD, the pathogenic protein α-Syn misfolds and aggregates to form Lewy bodies, which interrupt normal cellular processes and lead to neuronal death. So far, eight autosomal dominant mutation sites of α-Syn have been identified, providing promising targets for drug design [42,43]. The huntingtin protein, particularly in its mutated form, plays a significant role in the pathogenesis of certain NDDs, most notably HD. Its effects include the disruption of proteostasis, interference with transcriptional regulation, impairment of synaptic function, and contribution to mitochondrial dysfunction and oxidative stress [44]. Collectively, these pathologies underline the complexity of NDDs and highlight the critical need for targeted therapeutic strategies addressing the unique mechanisms of each abnormally folded protein.

### 2.5. Axonal Transport Defects

The formation, maturation, and uptake of axons are critical for the formation of synaptic connections between neurons, and many NDDs are associated with defects in neuronal axonal transport [7]. Tau is a microtubule-associated protein (MAP) that is abundant in the axonal cavities of neurons and plays a crucial role in regulating microtubule dynamics and organizing microtubule structure in axons and the growth cones of neuronal cells. When tau proteins are over-phosphorylated, they self-aggregates into neurofibrillary tangles (NFT), which block axon transport and cause neuronal death, as evidenced by the phosphorylation at S199 of tau protein leading to AD progression [45].

Furthermore, long-range axons are particularly susceptible to damage, and axonopathy is often an early hallmark of neurodegeneration [46]. For instance, the axons of dopaminergic neurons in the brain are particularly long, making them susceptible to disruption of axonal transport and damage in PD [47]. Motor neurons in the brain and spinal cord have long axons, which makes them heavily dependent on axonal transport in amyotrophic lateral sclerosis (ALS) [48]. In summary, axons play a crucial role in NDDs, and disruption of axonal transport is a common mainstay of many of these diseases.

### 2.6. Gene Mutations

Scientific studies have found that individuals carrying specific genes are more likely to develop NDDs, such as the APP gene in AD patients and the SNCA gene in PD patients. All of these risk genes are associated with one or more physiological processes, such as pathological protein aggregation and clearance, neuroinflammation, cellular metabolism, cellular immunity, and oxidative stress [49]. The exploration of gene mutations in the pathogenesis of neurodegenerative diseases has advanced significantly over the past decade. For instance, Shih et al. [50] investigated the electronic properties of DNA in disease-related genes, revealing notable differences in the electronic properties of pathogenic mutations compared to all other possible mutations, which suggests a potential role for these properties in cellular processes and disease etiology. Bordner and Zorman [51] utilized large-scale homology modeling of human protein complexes to detect non-neutral mutations, finding a higher prevalence of disease-associated mutations in various binding sites. This approach provided experimentally testable biochemical mechanisms for mutations in NDDs. A meta-analysis by Bayraktar et al. [52] revealed common pathological pathway patterns in AD, PD, and ALS, particularly in cellular heat stress response and γ-Aminobutyric Acid (GABA) synthesis pathways, with genes like APP and HTT showing unique variations in patients. Another comprehensive meta-study by Ruffini et al. [53] recruited data from 177 studies encompassing over one million patients to unravel shared genetic patterns in neurodegenerative diseases, The study revealed a significant number of shared differentially expressed genes, particularly at the transcriptomic and proteomic levels across all diseases, highlighting notable genetic interrelations, especially between AD and PD, and AD and ALS. Through gene ontology (GO) analysis, 139 genes were identified as consistently differentially expressed across several transcriptomic experiments on these diseases, implicating them in key neurodegeneration-related processes such as response to heat, hypoxia, cytokine regulation, angiogenesis, and RNA catabolic processes. The analysis further delineated a close genetic relationship between AD and HD. These studies collectively underscored the profound impact of gene mutations and expressions in the pathogenesis of neurodegenerative diseases, illuminating common underlying mechanisms and potentially paving the way for targeted therapeutic strategies.

### 2.7. Disorders in Gut Microbiota

The gastrointestinal system is home to trillions of GMS that have important physiological functions, including metabolism, nutrient absorption, and immune system changes, etc. [54]. Recent studies on the gut–brain axis (GBA) have found that, although the gut and brain are anatomically independent of each other, the gut microbiota (GM) can communicate with the central nervous system via multiple pathways, such as the endocrine system and circulatory system, etc. [55]. When the GM changes it may affect the intestinal barrier integrity, trigger neuroinflammation, interfere with neural mitochondrial function, and abrogate neurotransmitter synthesis, thereby driving the occurrence of NDDs [56].

Regulation of the GM can alleviate AD by reshaping microglia. Short chain fatty acids (SCFAs) produced by the GM can transfer from the gut to the systemic circulation and cross the blood–brain barrier, where they have different effects on the immune cells. Thus, delicate control of the GM composition will alter the repertoire of SCFAs in brain and polarize the maturation and function of microglia in favor of the patients [57]. In another case, Daniel et al. found that the immature phenotype of mouse microglia is epigenetically marked by H3K4me3 and H3K9ac, and these genes are related to increased mitochondrial mass and respiratory chain dysfunction. One of the reasons for such abnormality is the loss of intestinal peristaltic flora and the subsequent accumulation of acetic acid in the lesion, which can be reversed by GM modulation [58]. These findings suggest that the GM is able to regulate microglial phagocytosis and disease progression during neurodegeneration.

Braak et al. proposed the hypothesis that PD may originate in the gut and subsequently progress to the brain, as evidenced by α-Syn progressing from peripheral sites to the central nervous system through the vagus or olfactory pathways [59]. The hypothesis explained the early intestinal symptoms, such as movement disorders and inflammatory bowel disease, in many patients. α-Syn is considered an early biomarker of PD, while some SCFAs, such as acetate, propionate, and butyrate, can interrupt the development of neuroinflammation in PD through regulating α-syn aggregation. However, such beneficial SCFAs are produced in low quantity by the ill-defined GM from PD patients [60]. Therefore, partial neurotrophic and anti-inflammatory substances that may reduce neuroinflammation and the misfolding of α-Syn in the neural system are promising for the prevention and treatment of PD.

## 3. Potential Active Compounds from *Ganoderma lucidum* against Neurodegenerative Diseases

Clinical trials of drugs that specifically interfere with designated neurotoxic pathways have demonstrated some promising results in alleviating NDD-related symptoms. However, those medications are often administered long after the lesions have occurred in NDDs, when all of the factors contributing to neuronal death have been activated, due to the lack of early markers and late diagnosis. Therefore, versatile and multi-target complementary medicines as well as natural alternatives, have drawn great interest as NDD treatments because they may delay or halt the onset of NDDs with long-term administration [61].

*Ganoderma lucidum* is widely used in dietary ingredients and traditional medicine in Asia, and its extracts have been processed into a variety of healthy products for sedation and longevity applications [11]. Hitherto, researchers have extracted more than 400 bioactive compounds from the spores, mycelium, and fruiting bodies of *Ganoderma lucidum*, including triterpenoids, polysaccharides, proteins, amino acids, nucleosides, nucleic acids, steroids, lactones, fatty acids, organic germanium, selenium, enzymes, and alkaloids, etc. Some of these compounds demonstrate unique bioactivities that can be leveraged to abrogate the occurrence or alleviate the symptoms of NDDs [62]. For instance, components from GLE are able to diffuse from the bloodstream to the CNS, thereby overcoming the blood–brain barrier, and may become promising anti-neurodegenerative drug candidates [12,13,14].

### 3.1. Polysaccharides

*Ganoderma lucidum* polysaccharide (GLP) is one of the most important active ingredients of *Ganoderma lucidum,* with antioxidant, anti-inflammatory, and immune-enhancing effects which play an important role in the treatment of NDDs [63,64]. GLPs have a variety of biological activities, which are related to the composition of monosaccharides in polysaccharides, branching degree, the number of hydroxyl substitutions on branch chains, and relative molecular weights. So far, more than 200 fractions of polysaccharides have been isolated from *Ganoderma lucidum*, mainly composed of β-glucan [65]. Most GLPs are heteropolysaccharides with linear or branched molecules, molecular weights ranging from 2 to 800 kDa, and side chains containing glucose, galactose, mannose, fructose, xylose, or arabinose. These side chains are connected by β-(1→3), β-(1→4), or β-(1→6) linkages. The conformation of GLPs is generally a three-helix structure, which empowers their unique interaction features and specific biomarkers in the setting of disease progression [64].

One of the most prominent bioactivities of GLPs is their antioxidative effect, which closely relates to the pathogenesis of NDDs. Zheng et al. [66] adopted an ultrasonic assisted co-extraction (UACE) technique to extract polysaccharides from *Ganoderma lucidum*, resulting in optimal extraction rates of 0.63%. According to the results of 2,2-diphenyl-1-picrylhydrazyl (DPPH) scavenging experiments, the antioxidant capacity of polysaccharides obtained from the optimized UACE process is higher than that extracted by the traditional hot water extraction method. Xu et al. [67] extracted GLPs by the hot water extraction method. The crude polysaccharides were separated by a DEAE-52 cellulose column (1.6 cm D × 40 cm H) and further degraded by ultrasound to gain a new fraction of GLP (GLPUD). The SEM results showed that the molecular weight of GLPUD was 13.6 KDa and the viscosity was 29.88 mL/g. The contents of glucuronic acid and sulfate after GLPUD degradation were higher than those of conventional GLPs. In vivo experiments show that GLPUD has hypolipidemic and antioxidant properties, which can reduce the contents of lipid factors (Al, TC, TG and LDL-C) and MDA, and increase the contents of HDL-C, GSH-Px, and SOD in mice. In order to explore the effect of heat stress (HS) on the antioxidant capacity of GLP, Tan et al. [68] exposed harvested *Ganoderma lucidum* fruiting bodies to temperature control chambers at different temperatures (28, 37, 42 or 45 °C) and grinded them into powder after the incubation. Then the powder was extracted by hot water and deproteinized by the Savag method to harvest the total polysaccharides. The results showed that the antioxidant capacity of the polysaccharides isolated by HS was significantly improved. There were mainly four polysaccharide fractions in the HS group, with molecular weights of 0.5 × 10^4^, 56.4 × 10^4^, 133.5 × 10^4^, and 855.2 × 10^4^ Da. Compared with the blank group, the glucose content in the HS group was decreased by 22.73%, while the content of other five monosaccharides (mannose, rhamnose, ribose, xylose, and arabinose) was increased to varying degrees. Therefore, HS treatment can improve the antioxidant activity of GLP in vitro, which may be due to changes in its monosaccharide composition. These results suggest that GLP may be a potential treatment for NDDs caused by oxidative stress.

Some GLPs have been shown to have strong anti-inflammatory effects which could be employed to treat NDDs. Jia et al. [69] isolated and purified a water-soluble polysaccharide (GLP-2) from *Ganoderma lucidum* by the microwave-assisted freeze-thaw method. The results showed that GLP-2 was mainly composed of β-1, 3-glucose and β-1, and 6-glucose. The chain conformation of GLP-2 was analyzed by SEC-MALLS RI, which indicated a semi-rigid chain conformation. The molecular weight is 16.7 × 10^4^ Da and the molecular size is about 61.2 nm. Anti-inflammatory experiments showed that GLP-2 could inhibit the production of NO, TNF-α, IL-1β, and IL-6 in RAW 264.7 macrophages stimulated by LPS. Hu et al. [70] collected crude polysaccharides from the fruiting bodies of *Ganoderma lucidum* by hot water extraction. Afterwards, the purified GLP was obtained by gel filtration chromatography with Superdex-G 200 (GE Healthcare, Chicago, IL, USA). Such GLP is composed of glucose (72.5%), mannose (8.3%), galactose (5.7%), and glucuronic acid (13.5%), with a molar ratio of 4.91:1:28:0.71 and a molecular weight of 1013 kDa. This GLP both decreased the pro-inflammatory cytokine (TNF-a, IL-6, and IL-1β) secretion and ROS levels in LPS-stimulated inflammatory macrophages and inhibited COX-2 expression in enteric Caco-2/macrophage co-culture inflammatory models. Studies on molecular mechanisms suggested that it inhibited the activation of the LPS-induced MAPK signaling pathway and regulated oxidative stress by activating the Nrf2/Keapl signaling pathway.

Some GLPs are primary components responsible for the regulatory effects of *Ganoderma lucidum* on the immune system, hence contributing to a more regulated inflammatory response, which is crucial in slowing down or halting the progression of neurodegenerative diseases. Liu et al. [71] isolated and converged polysaccharides with molecular weights in the range of 1.75 × 10^3^~1.14 × 10^4^ g/mol from the fruiting bodies of *Ganoderma lucidum* by the hot water extraction method. The monosaccharide composition analysis showed that the polysaccharides were mainly composed of Glc (54.16~85.76%), Gal (4.88~39.40%), and Man (0.26~20.51%). The correlation analysis between molecular weights and the capacity to bolster immunity showed that polysaccharides with molecular weights of 4.27 × 10^3^~5.27 × 10^3^ and 1 × 10^4^~1.14 × 10^4^ g/mol were the main active fractions. High contents of Man, Gal, and GluA could further enhance the immune regulatory effects of GLP. In another case, Gokce et al. [72] tried to extract GLPs from the fruiting body with hot water and trichloroacetic acid. The resulting GLP showed 59.4% carbohydrate (55.35% D-glucose, 2.37% D-mannose, 1.68% D-galactose) and 30.33% protein, with an average molecular weight of 6.279 × 10^4^ Da. The 13C NMR CP-MAS signal of the GLP proved that its backbone was glycoprotein in the form of β-D-glucan. Animal experiments demonstrated that this GLP significantly alleviated spinal cord injury, as evidenced by histopathological scores, ultrastructural scores, and functional tests, as well as reduced the levels of apoptosis factors (caspase-3, TNF-α), inflammatory factors (MPO, NO), and the oxidative stress-related protein MDA in rats with traumatic spinal cord injury.

### 3.2. Triterpenoids

*Ganoderma lucidum* triterpenoids (GLTs) are the other main active components of *Ganoderma lucidum* besides polysaccharides, which biogenetically possess a chemical structure of 30 carbon atoms with A, B, C, and D tetracyclic skeleton and side chains. The molecular weights of GLTs typically range between 400 and 600 kDa, and their chemical structures are diversified, primarily attributable to variations in the sites and degrees of oxidation (See Table 1). Based on the terminal substituents of the side chains, GLTs are usually divided into ganoderic acid and ganoderol, which have carboxyl and alcohol hydroxyl groups at the ends of their side chains [73]. Ganoderic acid A (GAA) represents the most thoroughly researched among ganoderic acids derived from *Ganoderma lucidum* due to its effects against NDDs. For example, Ahmad et al. [74] investigated the potential of GAA against PD through a computational analysis involving ADMET analysis, molecular docking, molecular dynamics simulations, and MMGBSA calculations. Focused on the leucine-rich repeat kinase 2 (LRRK2), a key protein implicated in PD, the study concluded that GAA exhibited promising therapeutic effects necessitating further in vitro and in vivo validation.

Other than GAA, Liu et al. [75] separated triterpene ganoderic acid C1(GAC1) from *Ganoderma lucidum* by repeated silica gel, high-performance liquid chromatography, and sephadex LH-20 chromatography column. GACI blocked the activation of NF-κB and reduced the production of pro-inflammatory factors (TNF-a, IFN-g, and IL-17A) in RAW 264.7 macrophages in patients with Crohn’s disease. Heme oxygenase-1 (HO-1) is an enzyme that plays important anti-inflammatory and antioxidant roles in the human body. Choi et al. [76] isolated 12 lanostane triterpenes from the fruiting bodies of *Ganoderma lucidum*, among which butyl lucidenate E2, butyl lucidenate D2 (GT-2), butyl lucidenate P, butyl lucidenate Q, Ganoderiol F, methyl ganodenate J, and butyl lucidenate N significantly inhibited LPS-activated inflammatory responses in mouse macrophages and induced HO-1 expression. Notably, GT-2 induced the expression of HO-1 in both mRNA and protein levels through the PI3K/AKT-Nrf2 pathway and inhibited the secretion of anti-inflammatory factors (TNF-α, IL-6 and NO), and the expression of iNOS and COX-2 in RAW264.7 cells. Koo et al. [77] identified and isolated a novel ganoderma alkane triterpenoid compound, ganosidone A, featuring a unique four-membered ring system (e-ring) extending from C-1 to C-11, together with eight known derivatives of this compound, from methanol extracts of *Ganoderma lucidum* fruiting bodies. These compounds underwent anti-inflammatory testing, revealing that lucidumol A and ganodermanontriol notably inhibited NO production in LPS-induced RAW 264.7 cells, demonstrating the most significant effects among the tested compounds.

Another well-recognized characteristic of GLTs is their ability to neutralize harmful free radicals in the body. This action helps to prevent oxidative stress, a critical factor in the development of various diseases, including NDDs and cardiovascular diseases [78]. The accumulation and antioxidant activity of GLTs vary across different developmental stages of cultivation. Notably, the concentration of GLTs peaks in young fruiting bodies, while the GLTs extracted from the budding stage demonstrate the highest scavenging rates for DPPH, OH, and ABTS (2,2′-Azinobis-(3-ethylbenzthiazoline-6-sulphonate)) free radicals, as well as reducing the ROS level in LPS-induced macrophages [79]. Wang et al. [80] isolated seven secondary metabolites from the fruiting bodies of *Ganoderma lucidum*, including a new trichomeric triterpene called methyl ganoderate G1, and six known triterpenes, namely ganoderic acid D2, ganoderic acid H, ganoderiol B, ludidumol B, lingzhine E, and lingzhine F. The antioxidant capacity and neuroprotective activity of the isolated compounds against either H_2_O_2_ or aging-Aβ-induced SH-SY5Y cell death were evaluated in vitro. The results revealed that methyl ganoderate G1, lingzhi E, and lingzhi F exhibited the most potent antioxidant and neuroprotective effects. Furthermore, all isolated compounds underwent assessment for their free radical scavenging activities. Notably, lingzhi E and lingzhi F demonstrated substantial scavenging abilities in ABTS and ORAC (oxygen radical absorbance capacity) assays, indicating strong free radical scavenging potential.

**Table 1:** GLTs with potential anti-NDD effects.

No.	Compound Name	Structure	Reference
**1**	Ganoderic acid C1	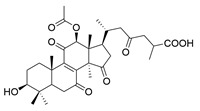	[75]
**2**	Ganoderiol F	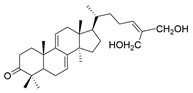	[76]
**3**	Ganodermanondiol	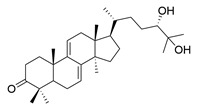	[76]
**4**	Lucidumol B	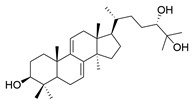	[76]
**5**	Methyl ganodenate J	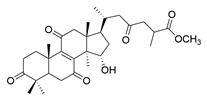	[76]
**6**	Methyl lucidenate A	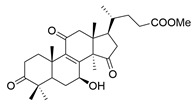	[76]
**7**	Methyl lucidenate N	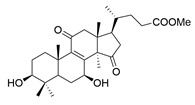	[76]
**8**	Butyl lucidenate D2	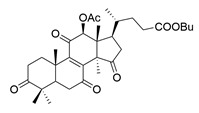	[76]
**9**	Butyl lucidenate E2	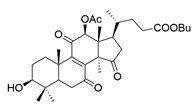	[76]
**10**	Butyl lucidenate H	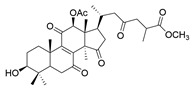	[76]
**11**	Butyl lucidenate N	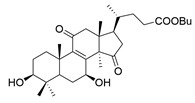	[76]
**12**	Butyl lucidenate P	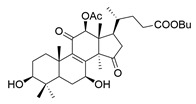	[76]
**13**	Butyl lucidenate Q	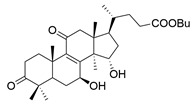	[76]
**14**	Ganoderic acid A	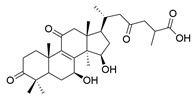	[77]
**15**	Methyl ganoderate A	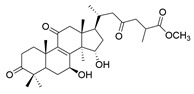	[77]
**16**	Methyl ganoderate H	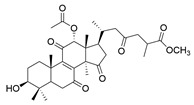	[77]
**17**	Ganosidone A	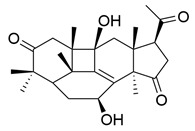	[77]
**18**	Ganodermanon triol	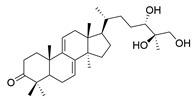	[77]
**19**	Ganolucidic acid A	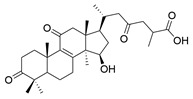	[76]
**20**	Ganolucidic acid E	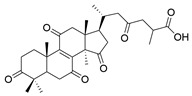	[77]
**21**	Lucidumol A	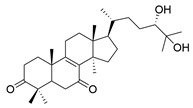	[77]
**22**	Lucidumol C	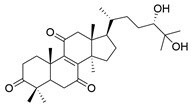	[77]
**23**	Ganoderic acid D2	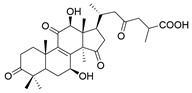	[80]
**24**	Ganoderic acid H	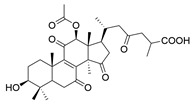	[80]
**25**	Ganoderiol B	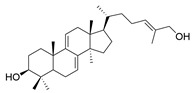	[80]
**26**	Lucidumol B	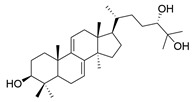	[80]
**27**	Lingzhine E	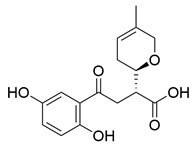	[80]
**28**	Lingzhine F	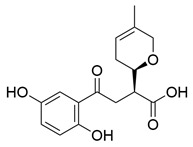	[80]

### 3.3. Proteins and Peptides

Historically, triterpenoids, polysaccharides, and other small molecules have been regarded as the primary bioactive components in *Ganoderma lucidum*. However, accumulative studies suggest that the protein components in *Ganoderma lucidum* also play a significant role as active molecules, expanding the understanding of its pharmacological profile. *Ganoderma lucidum* peptides, produced through hydrolysis using 2.5% (*w*/*v*) proteases (papain and pepsin-trypsin), demonstrated a potent scavenging capability against ABTS, DPPH, and NO free radicals. Notably, the component with a molecular weight of 0.65 kDa exhibited the strongest scavenging ability. Peptides with a lower molecular weight (<0.65 kDa) also showed significant antioxidant activity. These peptides were further fractionated using RP-HPLC. The fraction with the highest free radical scavenging activity was subsequently selected for amino acid sequencing via Q-TOF-LC-MS/MS. This process led to the identification of two novel peptide sequences, DRVSIYGWG and ALLSISSF, both exhibiting strong free radical scavenging properties. Synthetic versions of these peptides were found to have a cytoprotective effect on Caco-2 cells during cellular antioxidant activity (CAA) assessment. Additionally, they selectively inhibited pro-inflammatory cytokine genes (iNOS, COX-2, IL-6, and TNF-α) at the transcriptional level in RAW264.7 macrophages following LPS stimulation. This study underscores the potential of peptides derived from *Ganoderma lucidum* proteins in reducing oxidative stress and inflammation [81].

The gut–brain axis, identified as a therapeutic target for NDDs, is known to influence metabolic activity, neuroimmune function, and sensory neuronal signaling [82]. Xiong et al. [83] developed *Ganoderma lucidum* protease hydrolysate (GLPH) using *Ganoderma lucidum* as a substrate and pepsin and trypsin as enzymatic agents through in vitro biomimetic enzymatic hydrolysis. GLPH demonstrated cholesterol lowering effects, characterized by reductions in triglycerides, total cholesterol, low-density lipoprotein cholesterol, alanine aminotransferase, and aspartate aminotransferase levels, while increasing high-density lipoprotein cholesterol in serum and the liver. At the liver mRNA level, GLPH led to the downregulation of sterol regulatory element-binding transcription factor 1C and acetyl-coenzyme A carboxylase. Conversely, it upregulated AMP-activated protein kinase, acyl-CoA oxidase, farnesoid X receptor, and peroxisome proliferator-activated receptor α. The treatment with GLPH also modified the composition of intestinal flora, notably reducing the relative abundance of potentially harmful bacteria such as alistipes and clostridium. Spearman’s correlation analysis indicated a relationship between changes in fecal microbiota and biochemical markers. These findings suggest that GLPH can effectively improve lipid metabolism disorders and regulate the structure of intestinal microbiota, which may be beneficial to alleviating NDDs.

### 3.4. Other Components

*Ganoderma lucidum* contains a variety of bioactive compounds, other than polysaccharides, triterpenoids, and proteins, with diverse pharmacological activities. Though not directly linked with NDDs, some of these compounds may work on other related systems as complementary treatments. For example, adenosine derivatives derived from *Ganoderma lucidum* were found to have antioxidant effects, preventing the loss of cell viability and the production of ROS after H_2_O_2_ exposure [84,85]. Ganoderma alkaloids, such as Ganoderma amine B, arrest the MCF-7 cell cycle in the S-phase by inducing DNA fragmentation, thus reducing mitochondrial membrane potential, and have good antioxidant activity on MCF-7 cells (EC50 value is 0.27 ± 0.02 μmol/mL), which may benefit the nervous system [86]. Naringenin and hesperetin, found in *Ganoderma lucidum*, have been studied for their inhibition of the dengue virus. Their antiviral and anti-inflammatory properties could be beneficial for neurodegenerative diseases, where inflammation and potential viral links are areas of interest [87]. Albeit there are other bioactive compounds in *Ganoderma lucidum* that might possess neuroprotective effects and suggest potential therapeutic applications, the exact mechanisms of these compounds in the context of neurodegeneration need further exploration [88].

## 4. The Effects of *Ganoderma lucidum* on the Pathological Hallmarks of Neurodegenerative Diseases

Since the biological activities of *Ganoderma lucidum* on neural systems have been confirmed in many cases, its fruiting body along with or in combination with other medicines are expected to be used to treat NDDs in clinical practice. A phase Ⅱ interventional trial of *Ganoderma lucidum* was conducted to determine its effectiveness and safety in treating PD when combined with L-dopa (ClinicalTrials.gov, ID: NCT03594656). The study indicated that *Ganoderma lucidum* was safe, well tolerated, and improved symptoms as an add-on therapy to levodopa in early PD patients. Inspired by the results, the research group designed a multicenter, randomized, double-blind, placebo-controlled, delayed-start trial to evaluate the effects of *Ganoderma lucidum* on modifying disease progression in untreated PD patients (ClinicalTrials.gov, ID: NCT00224263). In order to guide the development and legitimate application of *Ganoderma lucidum*-based medications, massive studies on their therapeutic mechanisms against NDDs are ongoing and yet to be discussed in this section (Table 2).

### 4.1. Target on Pathogenic Proteins

Aβ and tau proteins, as well as the soluble oligomers of these two proteins, are hallmarks for AD, which can spread through different regions of the brain, directly causing synaptic dysfunction, neuronal damage, and cognitive decline in AD patients. Some studies have found that *Ganoderma lucidum* can neutralize the neurotoxicity of Aβ amyloid peptide in the setting of AD [89]. Lai et al. [90] extracted an aqueous fraction with hot water from the fruiting body of *Ganoderma lucidum* and demonstrated that *Ganoderma lucidum* significantly preserved synaptic density by maintaining synaptophysin levels, effectively counteracting the synaptotoxicity induced by Aβ. Moreover, *Ganoderma lucidum* markedly reduced neuronal apoptosis, as evidenced by the decreased number of apoptotic bodies and lower caspase-3-like activity in neurons treated with Aβ. This neuroprotective action was further supported by *Ganoderma lucidum’*s ability to attenuate the activation of stress kinases, including JNK, phosphorylated c-Jun, and p38 MAPK, pathways commonly associated with apoptotic signaling in AD. These findings highlighted the capacity of *Ganoderma lucidum* to mitigate key pathological features of AD, such as synaptic degeneration and neuronal apoptosis, by modulating critical pathogenic proteins. A Morris’s water maze test by Yu et al. [91] found that GLTs could significantly alleviate cognitive impairment in AD mice as well as improve their spatial learning and memory. Histological analyses indicated that GLTs preserved hippocampal tissue integrity and reduced NFTs formed by the hyperphosphorylation of the tau protein in neurons. A critical aspect of the study is the observed reduction in neuronal apoptosis and oxidative damage, achieved through the inhibition of the ROCK signaling pathway, as evidenced by changes in the expression of key apoptosis- and antioxidative-related proteins. Furthermore, in vitro experiments reinforced GLTs’ protective effects on hippocampal neurons, highlighting their potential in countering AD-induced oxidative stress and neuronal damage. Cui et al. [92] investigated the neuroprotective capacities of GAA and ganoderic acid B (GAB) against okadaic acid (OA)-induced cytotoxicity within PC12 cellular models. Both GAA and GAB significantly attenuated tau hyperphosphorylation at pivotal sites (S199 and T231), implicated in the pathogenesis of AD, with GAB demonstrating a notably enhanced efficacy. The research elucidated the underlying protective mechanism, accentuating GAB’s capacity to diminish the phosphorylation of glycogen synthase kinase-3β (GSK-3β) at Tyr216, a principal kinase involved in tau phosphorylation. Therefore, GAB may become a potent modulator against NDDs related to tau hyperphosphorylation. Zhao et al. [93] conducted a study on sporoderm-deficient *Ganoderma lucidum* spores (GLSs) which revealed their significant impact on pathogenic proteins associated with AD. Specifically, GLS treatment in a rat model of AD induced by streptozotocin (STZ) led to a notable reduction in the expression of Aβ protein. Additionally, the same treatment was effective in reversing STZ-induced increases in tau protein expression and its phosphorylation at Ser199, Ser202, and Ser396. Given that P-tau is a hallmark of AD, associated with neurofibrillary tangles and characteristic disease features, the ability of GLS to influence tau phosphorylation underscores its potential as a therapeutic agent for modulating tau pathology in AD.

Microtubule affinity regulation kinase 4 (MARK4) overexpression has been observed in the initiation of neurodegenerative diseases, such as AD and PD. Overexpressed MARK4 is responsible for the phosphorylation of tau proteins at the Ser262 site, which is required for the binding of microtubules to tau proteins. Thus, potent MARK4 inhibitors may be promising drug candidates for NDDs [94]. Based on that hypothesis, Ahmad et al. [95] employed molecular docking and dynamics simulations to identify potent inhibitors of MARK4 from *Ganoderma lucidum*. Among the five analyzed compounds, GAA and ganoderenic acid B emerged as the most promising ones, exhibiting high binding affinity and stable interactions with MARK4, a protein involved in the phosphorylation of tau proteins. Their stability and interactions were validated through molecular dynamics simulation and MMGBSA (molecular mechanics generalized born surface area) calculations. The computational validation of these compounds as potential AD therapeutics underscored the need for further in-depth preclinical and clinical evaluations. Qi et al. [96] revealed the significant role of GAA in promoting Aβ clearance and mitigating cognitive deficiencies associated with AD. GAA was found to facilitate Aβ_42_ degradation in microglial cells, primarily through the autophagy pathway, as evidenced by experiments using inhibitors of amyloid-degrading enzymes. This effect is mediated via the activation of the autophagy pathway through the Axl receptor tyrosine kinase and RAC/CDC42-activated kinase 1 (Pak1) signaling pathway, with increased phosphorylation of Axl and Pak1 and conversion of LC3B-I to LC3B-II in GAA-treated cells. The study extended these findings to an in vivo AD mouse model, where GAA treatment not only ameliorated cognitive deficiencies but also reduced Aβ_42_ levels in the hippocampus. This was further corroborated by behavioral tests like object recognition and Morris’s water maze tests, demonstrating GAA’s efficacy in restoring learning and memory abilities impaired by Aβ injection. The study concludes that GAA enhances autophagy in microglial cells by augmenting Axl phosphorylation, thereby promoting Aβ_42_ clearance and offering potential therapeutic value in treating AD. Shen et al. [97] demonstrated that GAA could effectively counteract the deleterious effects of Aβ_25–35_, a neurotoxic subtype of Amyloid-β, by enhancing cell viability and reducing apoptosis and senescence in AD model cells. Meanwhile, GAA was found to inhibit the downregulation of autophagy-related proteins like ATG5 and Beclin 1, thereby alleviating autophagy impairments characteristic of AD. Central to this process is the role of peptidyl arginine deiminase 4 (PADI4), a mediator of autophagy, which operates through the Akt/mTOR pathway to delay cellular senescence. The study uncovered a synergistic effect of GAA and PADI4, suggesting that the combination can enhance cell viability while mitigating senescence in AD models. This indicated GAA’s potential not only for modulating key pathological processes of AD but also in influencing the progression of the disease by targeting the Akt/mTOR signaling pathway. Li et al. [98] isolated and evaluated compounds from *Ganoderma lucidum* capable of disintegrating Aβ fibrils. Using a ligand fishing method with Aβ_42_ fibril-immobilized magnetic beads, six compounds—ganodermanontriol, ganoderic acid DM, ganoderiol F, ganoderol B, ganodermenonol, and ergosterol—were identified as Aβ_42_ fibril disintegrators. In vitro and in vivo assessments revealed that these compounds not only significantly mitigated Aβ_42_-induced neurotoxicity by enhancing cell viability and reducing LDH release but also demonstrated strong neuroprotective effects, particularly ergosterol and ganoderic acid DM, in alleviating cognitive dysfunction and hippocampal neuron loss in mouse models. The protective activities of these compounds were further attributed to their ability to inhibit neuron apoptosis and oxidative stress, mediated through the activation of the Nrf2-Keap1 signaling pathway. The above findings suggested that *Ganoderma lucidum* may help to protect neurons from the destructive effects of some pathogenic proteins, thus suggesting a potential therapeutic role for NDDs.

### 4.2. Regulation of Inflammatory Factors

Microglia-mediated neuroinflammation is a hallmark of NDDs. In these diseases, activated microglia produce a variety of inflammatory mediators, which promote neuronal damage and disease progression. Zhang et al. [99] provided compelling evidence of the neuroprotective role of *Ganoderma lucidum* in PD, highlighting its efficacy in inhibiting microglial activation and the resultant neuroinflammation. The research showcased that GLE significantly curbed the production of microglia-derived proinflammatory and cytotoxic factors, including nitric oxide, TNF-α, and IL-1β, in a dose-dependent manner, thereby mitigating the key drivers of neuronal damage in PD. Also, GLE down-regulated TNF-α and IL-1β at the mRNA level, suggesting a deep-rooted genetic influence in its mechanism of action. The study further revealed GLE’s protective capabilities against methyl-4-phenylpyridine (MPP^+^)-induced dopaminergic neurodegeneration, demonstrating its direct neuroprotective impact irrespective of the presence of microglia. This implied that *Ganoderma lucidum* prevented neurodegeneration and restored neuronal function by dampening microglial activation and modulating inflammatory pathways. A recent study conducted by Hilliard et al. [100] demonstrated that GLE pretreatment effectively reduced NO release from activated BV-2 cells and downregulated the pro-inflammatory cytokines granulocyte-colony stimulating factor (G-CSF), IL1-α, monocyte chemotactic protein-5 (MCP-5), and macrophage inflammatory protein 3-α (MIP3-α) expression. In RT-PCR experiments, GLE preconditioning reduced the expression of mRNA involved in the translocation of NF-κB to the nucleus and the release of pro-inflammatory cytokines. It is evident that the preventive effect of *Ganoderma lucidum* on LPS-induced inflammation in BV-2 microglia is related to the NF-κB and MAPK signaling pathways. Cai et al. [101] indicated that GLP attenuated inflammation-associated microglia migration, morphological alterations, and phagocytosis, down-regulated the expression of pro-inflammatory cytokines (IL-1β, IL-6, and iNOS), and promoted the expression of anti-inflammatory cytokines (TGF-β). These effects are associated with the expression of MCP-1 and C1q. On the other hand, GLT reversed the cognitive dysfunction of aged rats induced by D-galactose, decreased the levels of pro-inflammatory factors NO and TNF-α, increased the level of anti-inflammatory factor IL-2, and inhibited the expression of PI3K/AKT/mTOR, thereby abrogating the inflammatory response during neurodegeneration [102]. An in vitro study revealed that GAA significantly inhibited LPS-induced proliferation and activation of BV2 microglia in vitro, facilitated the conversion of BV2 microglia from the M1 state to the M2 state, and inhibited the release of pro-inflammatory factors in BV2 microglia. Such inhibitory effects of GAA on neuroinflammation were mediated by the downregulation of the farnesoid-X-receptor (FXR) [103]. Further in vivo experiments showed that GAA improved cognitive ability, protected mitochondrial function, and regulated the JAK/STAT signaling pathway induced by the Th17/Tregs axis, thereby ameliorating neuroinflammation in AD mice [104]. Sheng et al. [105] isolated deacetyl ganoderic acid F (DeGA F) from the fruiting bodies of *Ganoderma lucidum* and investigated its effects on LPS-induced neural inflammation. As a result, it inhibited microglia and astrocyte activation by decreasing the production of NO and pro-inflammatory cytokines (TNF-α and IL-6), the mechanism of which related to the interruption of the NF-κB pathway. Kou et al. [106] discovered a new compound with anti-inflammatory effects, namely ganoderterpene A, from *Ganoderma lucidum*. This compound exhibited a pronounced ability to mitigate inflammation by effectively inhibiting nitric oxide production in LPS-stimulated BV-2 microglial cells. The anti-inflammatory action of ganoderterpene A is attributed to its suppression of key signaling pathways, specifically the MAPK and TLR-4/NF-κB pathways. Chen et al. [107] explored the efficacy of a fungal immunomodulatory protein derived from *Ganoderma* microsporum (GMI) in neuroprotection and neuroinflammation mitigation. The research demonstrated that GMI significantly reduced neuronal death and inflammatory mediator production, including NO, TNF-α, IL-1β, and prostaglandin E2 (PGE2), in rodent primary neuron/glia cultures. A key aspect of GMI’s mechanism is its ability to inhibit microglial activation, a crucial factor in neuroinflammation. This inhibition was accomplished through the downregulation of various inflammatory pathways, involving the suppression of NF-κB, AP-1, cAMP response element-binding protein (CREB), and Stat1 transcriptional activities and their upstream activators. Additionally, GMI aided in resolving oxidative stress and preserving protein phosphatase activities, further contributing to its anti-inflammatory effects. These findings positioned GMI as a promising candidate for addressing neuroinflammation-related neurodegenerative diseases.

### 4.3. Antioxidative Effects

The antioxidant effects of *Ganoderma lucidum* have been confirmed to protect neural systems from oxidative stress, thus being responsible for alleviating NDDs in many cases. Such effects are attributed to the chemical diversity of *Ganoderma lucidum*, including polysaccharides, polyphenols, triterpenes, aromatic meroterpenoids, and some other components [62]. These compounds constitute a crucial arsenal in strategies designed to combat the neurodegeneration associated with oxidative stress. A study by Zhang et al. [108] highlighted the significant antioxidative and anti-inflammatory properties of *Ganoderma lucidum* in the context of cerebral ischemia/reperfusion injury in rats. Administration with the hot water extract of *Ganoderma lucidum* reduced neuronal loss and malondialdehyde content in the hippocampus and serum, indicative of decreased lipid peroxidation. Additionally, a decrease in pro-inflammatory cytokines (TNF-α and IL-8) in the hippocampus and an increase in SOD activity, both in the hippocampus and serum, were observed. These results collectively underscored the prominent role of *Ganoderma lucidum’*s antioxidant effects in mitigating cerebral ischemic injury, offering insights into its therapeutic potential for NDD treatment. Yang et al. [109] explored the neuroprotective properties of *Ganoderma lucidum* water extract (GW) and its fermented form (FGW) by *Bifidobacterium bifidum* and *Lactobacillus sakei subsp. sakei*. The study highlighted that both GW and FGW inhibited AChE activity and exhibited strong neuroprotective activities against oxidative stress in PC12 cells, evidenced by enhanced cell viability and reduced lactate dehydrogenase release. Moreover, these extracts were effective in reducing H_2_O_2_-stimulated apoptosis and caspase-3 activity, underlining their potential caspase-dependent neuroprotective mechanisms. Ren et al. [13] demonstrated that GLE significantly improved motor performance and increased the survival of dopaminergic neurons in MPTP-treated mice. In neuro-2a cells, GLE was found to protect against MPP^+^-induced cell death, mitochondrial dysfunction, and oxidative stress by maintaining mitochondrial membrane potential, reducing reactive oxygen species, and preventing ATP depletion. The study also highlighted GLE’s role in modulating autophagy and apoptosis, potentially through the activation of AMPK/mTOR and PINK1/Parkin signaling pathways.

Polysaccharides are one of the most extensively studied antioxidant components in *Ganoderma lucidum*. When GLPs are ingested by the human body, they are rapidly absorbed, thereby increasing the total antioxidant activity in plasma [10]. A study conducted by Guo et al. [110] delineated that GLP significantly neutralized the neurotoxicity of MPP^+^, diminished Rot-induced degeneration of dopaminergic neurons, improved neuronal survival, increased mitochondrial complex I activity and mitochondrial membrane potential (ΔΨm), and decreased ROS levels, suggesting that GLP may treat NDDs through antioxidant effects. Li et al. [111] revealed the protective role of GLP against oxidative stress and apoptosis in H_2_O_2_-induced SH-SY5Y cells, primarily through the enhancement of mitochondrial function. Treatment with these polysaccharides not only increased ΔΨm and SOD activity, indicative of improved mitochondrial health and enhanced antioxidant defense, but also significantly reduced apoptotic rate and oxidative stress markers, such as MDA levels. Furthermore, the polysaccharides modulated apoptosis regulatory proteins by decreasing Bax and Caspase-3, as well as increasing Bcl-2 expressions, alongside promoting mitochondrial fusion over fission by downregulating fission proteins (Fis1 and p-Drp1) and upregulating fusion proteins (OPA1, Mfn1, and Mfn2). These findings collectively underscore the therapeutic potential of GLP in mitigating mitochondrial dysfunction and fostering cellular resilience in neurodegenerative disease contexts.

GLTs have also been reported to possess strong antioxidant activities which relate to neuroprotective effects. In one study, Wang et al. [80] isolated and characterized of seven secondary metabolites from the *Ganoderma lucidum* mushroom. Among these, a new lanostane triterpene (compound **1**, Methyl ganoderate G1), alongside known triterpenes and aromatic meroterpenoids, was identified using chromatographic and spectroscopic techniques. Notably, compounds **1**, **6** (Lingzhine E), and **7** (Lingzhine F) demonstrated significant antioxidant and neuroprotective activities, with compounds **6** and **7** showing radical scavenging activities comparable to positive drugs in ABTS and ORAC assays. The study underlined the potential of these compounds, particularly the meroterpenoids, as functional food ingredients for combating oxidative stress and preventing neurodegenerative diseases, laying a foundation for future bioactive research and practical applications in antioxidation and anti-neurological disease therapy. Wang et al. [102] revealed that dietary intervention with GLT significantly ameliorated oxidative stress markers in aged rats, evidenced by a decrease in serum levels of MDA, advanced glycation end products (AGEs), and NO. Concurrently, there’s an upsurge in the activity of crucial antioxidative enzymes, including T-AOC, GSH-Px, T-SOD, and CAT. Notably, the study underlined the role of GLT in modulating molecular pathways linked to oxidative stress, such as downregulating pro-inflammatory cytokines and upregulating key proteins like FOXO4 and SIRT1, integral to cellular antioxidative responses.

Zhou et al. [112] conducted a series of experiments on the neuroprotective role of GLS against cognitive decline and hippocampal damage caused by intracerebroventricular streptozotocin (ICV STZ) in rats. Preadministration of GLS, especially at a high dose of 8.0 g/kg, was found to significantly mitigate oxidative stress in the hippocampus, as evidenced by decreases in MDA, GSH, ATP and cytochrome oxidase (CytOx) levels, as well as an increase in glutathione reductase (GR). The reduction in oxidative markers coincided with improved cognitive functions, demonstrated in Morris’s water maze tasks, and a reduction in neuron apoptosis in the hippocampus, particularly in the CA3 region critical for learning and memory. The findings in this paper emphasized GLS’s potential in countering oxidative stress and mitochondrial dysfunction, thereby offering a promising avenue for treating neurodegenerative conditions like Alzheimer’s disease. Wu et al. [113] obtained the flavonoid extract of *Ganoderma lucidum* (GLFE) with an optimized preparation method and evaluated its anti-oxidative effects in PC-12 cells exposed to H_2_O_2_. Prominently GLFE enhanced cell viability by 13.76% compared to H_2_O_2_ treatment and elevated the activities of key antioxidant enzymes such as SOD, CAT, and GSH-Px in PC-12 cells, indicating its capacity to bolster cellular antioxidant defenses. Furthermore, GLFE upregulated the expression of PI3K and Akt, while downregulating Caspase-3, contributing to its protective effects against oxidative stress. All these findings underscore *Ganoderma lucidum’*s potential as an important antioxidant source for health food products and therapeutic applications in the treatment of NDDs.

### 4.4. Anti-Aging Effects

Aging, a natural process of gradual deterioration of biological functions in mature organisms, is a major risk factor for NDDs. It influences the development and progression of neurodegenerative diseases in multifaceted ways, among which redox dysregulation and elevated neuroinflammation are highlighted [114,115,116,117]. Chronic neurodegeneration is often accompanied by systemic/local inflammatory processes and NDDs [118]. As the body ages, the body’s immune system changes and older adults are more susceptible to inflammatory infections, which leads to a gradual accumulation of neuroinflammation and a risk of cognitive decline and memory impairment. Immune aging is thought to be directly related to brain aging and memory loss [119]. This aging-related neurodevelopmental disorder may be driven by proinflammatory cytokines produced by senescent cells in glial cells and other tissues, and it significantly affects the blood–brain barrier and central nervous system development [120]. Moreover, aging allows the accumulation of misfolded pathogenic proteins to reach pathological thresholds, triggering neuronal damage and leading to age-related mitochondrial dysfunction and increased ROS production. As in the case of AD, oxidative stress stimulates the expression of inflammatory genes and impairs the normal function of astrocytes, thus disrupting the blood–brain barrier and triggering abnormal Aβ deposition. With aging, the body’s antioxidant capacity decreases, which further aggravates the abnormal deposition of extracellular Aβ [121]. Therefore, anti-aging substances may become promising therapeutic agents for the treatment of NDDs. Zeng et al. [122] discovered that long-term administration of GLTs significantly mitigated age-associated brain physiological decline in mice, primarily through the enhancement of autophagy, regulation of sphingolipid metabolism, and prolongation of telomere length. The efficacy of GLTs extended beyond improving pathological features common in aging, such as cataract formation, hair loss, and skin relaxation, to enhancing the functionality of vital organs like the kidneys, spleen, and liver. A pivotal finding of the study was the upregulation of autophagy markers, especially LC3A/B, which suggested that GLTs could promote the autophagic clearance of harmful cellular metabolites, integral for maintaining neuronal health. Moreover, GLTs’ intervention was found to adjust the levels of sphingolipid metabolites, including sphinganine 1-phosphate, sphinganine, sphingosine 1-phosphate (S1P), and sphingosine, signifying its regulatory impact on sphingolipid metabolism, which is essential for the removal of pathological metabolites and the maintenance of cellular homeostasis in the brain. Weng et al. [123] discovered two novel ergosterol derivatives, ganodermasides A and B, from the spores of *Ganoderma lucidum*, which significantly extended the replicative lifespan of Saccharomyces cerevisiae by modulating the UTH1 gene. These compounds, featuring a unique hydroxylation at C-15, demonstrated anti-aging activity comparable to resveratrol, a known anti-aging substance, particularly at a concentration of 10 µM. The UTH1 gene’s involvement in aging and stress resistance pathways suggested that its modulation by ganodermasides could offer insights into the development of interventions for neurodegenerative conditions such as Alzheimer’s disease, where aging is a critical risk factor. Okoro et al. [124] explored the potential of Methyl Ganoderate E (MGE), a compound derived from the *Ganoderma lucidum* mushroom, in addressing aging-related neurodegenerative disorders through its impact on the model organism *Caenorhabditis elegans* (*C. elegans*). MGE extended the lifespan of *C. elegans* by 26% and significantly reduced the aggregation of the key neurodegenerative markers α-Syn and Aβ, suggesting a direct impact on mechanisms underlying PD and AD. These effects were mediated through the daf-16, hsf-1, and skn-1 pathways, associated with longevity and stress resistance, indicating MGE’s role in enhancing cellular stress responses and detoxification. Furthermore, transcriptome analysis revealed MGE’s promotion of proteolysis and neural activity, while inhibiting cell death processes, aligning with the insulin signaling pathway’s known roles in neuroprotection and disease management.

DNA methylation, recognized as one of the earliest identified epigenetic modifications in genes, plays a crucial role in modulating gene expression. This modification leads to physiological alterations that are closely associated with aging and significantly contributes to the development of age-related NDDs [125]. Lai et al. [126] identified that alcohol extracts from *Ganoderma lucidum* could regulate DNA methylation, thus delaying AD in rodent models. By administering 100 mg/kg/day of D-galactose to induce accelerated aging, the research demonstrated that these extracts up-regulated methylation regulators such as histone H3, DNMT3A, and DNMT3B in brain tissues and improved learning and memory function, reduced neuronal apoptosis, and down-regulated AD markers like Aβ_1–42_. The study concluded that compounds like ganoderic acid and lucidone A in *G. lucidum* are crucial in mediating these effects, offering potential therapeutic value in delaying AD through epigenetic modifications.

### 4.5. Rejuvenating Neurogenesis

Neurotrophins are a family of proteins that play a critical role in the survival, development, and function of neurons. The neurotrophin family consists of four members: nerve growth factor (NGF), brain-derived neurotrophic factor (BDNF), neurotrophin 3 (NT3), and NT4/5, which play an important role in the development, differentiation, and survival of neurons through activation of Trk receptors. Their significance in preventing neurodegenerative diseases lies in their ability to support neuronal health, promote neurogenesis, and enhance synaptic plasticity, which is crucial for learning and memory [127,128]. *Ganoderma lucidum* can stimulate or mimic the production of neurotrophic factor, thereby protecting neurons from neurotoxin-induced death. Cheung et al. [129] found that GLE can activate the extracellular signal-regulated kinases (ERKs) within the Ras/ERK signaling pathway, which plays a crucial role in regulating the growth and differentiation of PC12 cells, a model for studying neurodegenerative diseases. The activation of ERKs by GLE ensured the survival of neurons dependent on NGF, even at very low concentrations, by facilitating the differentiation of PC12 cells into dopaminergic neurons. More importantly, GLE contained neuroactive compounds that not only prevented apoptosis in NGF-depleted PC12 neurons but also induced their differentiation into neurons, as evidenced by the expression of phosphorylated neuronal markers, including 200 kDa neurofilaments (NF-H-P) and 160 kDa neurofilaments (NF-M-P). GLE triggered the phosphorylation of ERK1/2 and CREB in PC12 cells, indicating that GLE’s effects were mediated through both the Ras/ERK and CREB signaling pathways. A study by Ling-Sing Seow et al. [130] unraveled the NGF-like biological activity of *Ganoderma lucidum* in rat pheochromocytoma cells. Immunofluorescence staining showed that MEK/ERK1/2 and PI3K/Akt inhibitors had an effect on neuronal morphology, and in contrast to the negative controls, where the cells were relatively small and round with almost no visible neurites, the cells of the GW group became larger and longer, showing neurite elongation that was twice the diameter of the cell body. However, inhibitors of the MEK/ERK1/2 and PI3K/Akt pathways blocked the neurogenic activity of GLWE, resulting in atrophy and roundness of the cell bodies without significant neurite extension. These results indicated that *Ganoderma lucidum* promoted the neurite growth and differentiation of PC-12 cells by activating the MEK/ERK1/2 and PI3K/Akt signaling pathways. NPCs are self-renewing pluripotent cells with the ability to self-renew and differentiate into various types of neural cells, providing a promising avenue for neuroregeneration research. NPCs are the progeny of stem cell divisions, which usually undergo a limited number of replication cycles in vivo. Evidence suggests that promoting the proliferation of NPCs, which are involved in the repair of nerve damage, can alleviate AD-related cognitive decline, and is therefore considered a potential therapeutic route for AD [131]. Experiments by Huang et al. [132] have shown that oral administration of GLPs can promote NPC proliferation and neurogenesis in APP/PS1 transgenic AD mice, alleviate cognitive deficits, and reduce Aβ amyloid deposition. The polysaccharides were found to potentiate the activation of fibroblast growth factor receptor 1 (FGFR1) and its downstream signaling pathways ERK and AKT, suggesting a mechanism through which *Ganoderma lucidum* exerts its effects. While the burden of NDDs on society continues to increase, advances in cell therapy and the discovery of neurotrophic factors offer promising avenues for future treatments.

### 4.6. Other Roles

NO has multiple functions, including neurotransmission and immune response. In the nervous system, NO is produced by nitric oxide synthase (NOS). In NDDs, the activity of NOS may be affected, leading to changes in the level of NO. Excess NO causes extensive damage to the nervous system [133]. Yu et al. [134] revealed that GAA significantly attenuated sodium nitroprusside (SNP)-induced cytotoxicity and NO increase in SH-SY5Y cells but did not show the same protective effect in PC12 cells. Pretreatment with GAA resulted in significantly higher adrenaline content in SH-SY5Y cells compared to PC12 cells. Intriguingly, the addition of adrenaline significantly improved GAA’s protective effect in PC12 cells, which lack the ability to produce adrenaline, suggesting that adrenaline’s presence is crucial for GAA’s protective action. Moreover, the protective effect of GAA was blocked by either the β1-adrenergic receptor antagonist or the β2-adrenergic receptor antagonist in SH-SY5Y cells, indicating that GAA’s neuroprotective effect is mediated through β adrenergic receptors. These findings indicated GAA’s potential as a neuroprotective agent through the activation of β-adrenergic receptors, which may play a crucial role in mitigating NO-induced neural damage.

AChE plays a pivotal role in the nervous system, primarily by facilitating the breakdown of acetylcholine. In NDDs, AChE activity diminishes, leading to an impairment in the degradation of acetylcholine. This disruption significantly affects the transmission of nerve signals. The prevailing treatment strategy for AD aims to enhance cholinergic neurotransmission. Interestingly, *Ganoderma lucidum* has demonstrated anti-AChE properties, positioning it as a potential pharmacological candidate for AD treatment [135]. Lee et al. [136] isolated two new trichosteroidal triterpenes from the fruiting bodies of *Ganoderma lucidum*, named methyl *Ganoderma* A ethyl ester and n-butyl *Ganoderma lucidum* H, and obtained 16 known compounds. All the compounds showed excellent AChE inhibition activity, with IC50 values ranging from 9.40 uM to 31.03 μM. These results suggest that these lanostane triterpenes are promising inhibitors of AChE. Inspired by previous studies, Wei et al. [137] characterized 45 lanostane-type triterpenoids from *Ganoderma lucidum*, spotlighting two novel compounds, namely ganodernoid B2 and ganoderlactone G. The authors then investigated the inhibitory impact of these triterpenoids on AChE. Remarkably, compound **2** (11β-hydroxy-3,7-dioxo-5α-lanosta-8,24(*E*)-dien-26-oic acid) emerged as a potent inhibitor, exhibiting an IC50 value of 10.8 μM and a Ki of 14.95 μM, suggesting competitive inhibition kinetics, as evidenced by Lineweaver–Burk plot analysis. Docking analyses further elucidated the critical role of the C-17 side chain of ganoderic acid, particularly the 25-COOH group, in AChE inhibition, indicating a significant potential for therapeutic application in AD treatment. Luo et al. [138] uncovered five novel meroterpenoids from *Ganoderma lucidum*, among which dayaolingzhiols A and B were revealed to possess a unique 6/6/6 ring system, whereas dayaolingzhiols C–E featured a γ-lactone motif. Notably, dayaolingzhiols D and E exhibited potent AChE inhibitory activities, with IC50 values of 8.52 ± 1.90 μM and 7.37 ± 0.52 μM, respectively. Hasnat et al. [139] creatively grew the fruiting body of *Ganoderma lucidum* on brown rice (GLBR) and successfully elevated its antioxidant properties and inhibitory effects on AChE. Notably, GLBR extract showed significant AChE inhibition, with activity ranging from 19.46% to 57.01% across concentrations from 0.125 to 2.00 mg/mL. The IC50 value was determined to be 1.01 mg/mL which is comparable to that of tacrine, a positive control used in the study. The study also highlighted a strong correlation between the AChE inhibitory activity and the total phenolic and flavonoid contents of the extract, suggesting that the phenolic compounds, particularly quercetin and ursolic acid, which are abundant in GLBR, play a significant role in its neuroprotective potential.

**Table 2:** Effects of *Ganoderma lucidum* on the hallmarks of NDDs.

Mechanism	*Ganoderma lucidum* Ingredients	Model	Factors Related to NDDs	Potential Mechanism (↓: Upregulate, ↑: Downregulate)	Year/ References
Antipathogenic protein	GAA	BV2 microglial cells/Rats (Aβ_42_)	AD	Aβ_42_ ↓	2021 [96]
Antipathogenic protein	GAA/ganoderenic acid B	Molecular docking and dynamics simulation	AD	high binding affinity and stable interactions with MARK4	2022 [95]
Antipathogenic protein	GAA/GAB	PC12 cells (okadaic acid)	AD	tau hyperphosphorylation at S199 and T231 ↓	2023 [92]
Antipathogenic protein	ergosterol and ganoderic acid DM	SH-SY5Y cells (Aβ_42_ fibrils) C57BL/6 mice (Aβ_42_ fibrils)	AD	cell viability ↑ LDH level ↓ Cognitive dysfunction ↓ hippocampus neuron loss ↓ Nrf2-Keap1 signaling pathway ↑	2024 [98]
Antipathogenic protein	MGE	C. elegans	AD/PD	α-Syn, Aβ ↓	2024 [124]
Anti-neuroinflammation	GLE	microglia (LPS/MPP^+^)	PD	pro-inflammatory cytokines: NO, TNF-α, IL-1β ↓	2011 [99]
Anti-neuroinflammation	GLE	BV2(LPS)	Neuroinflammation	pro-inflammatory cytokines: G-CSF, Il-1α, MCP-5, MIP3α ↓ (NFB signaling)	2020 [100]
Anti-neuroinflammation	GLP	BV2 and primary mouse microglia (LPS/(Aβ_42_)	AD	Anti-inflammatory cytokines: TGF-β ↑ pro-inflammatory cytokines: IL-1β, IL-6, iNOS, MCP-1 ↓	2017 [101]
Anti-neuroinflammation	GLT	Rats (D-galactose)	Cognitive impairment	Anti-inflammatory cytokines: IL-2 ↑ pro-inflammatory cytokines: NO, iNOS, TNF-α, IL-6 ↓ (PI3K/AKT/mTOR pathway)	2020 [102]
Anti-neuroinflammation	DeGA F	BV2 Microglia/zebrafish embryos/mice model (LPS)	Neuroinflammation	Anti-inflammatory cytokines: IL-10 ↑ pro-Inflammatory Cytokines: NO, iNOS, TNF-α, IL-6, IL-1β ↓ (NF-κB Pathway)	2019 [105]
Anti-neuroinflammation	GAA	BV2 Microglia (LPS)	Neuroinflammation	M1 and M2 cell surface markers: iNOS ↓, Arg-1 ↑ Pro-inflammatory Cytokines: IL-1β, IL-6 and TNF-α ↓	2021 [103]
Anti-neuroinflammation	GAA	BALB/c Mice (D-galactose)	AD	pro-inflammatory Cytokines: IL-17A, IL-17F, IL-21, and IL-22 ↓ Anti-inflammatory cytokines: TGF-β1, IL-10, and IL-35 ↑ (inhibition of the JAK/STAT signaling pathway by Regulating the Imbalance of the Th17/Tregs Axis)	2021 [104]
Anti-neuroinflammation	Ganoderterpene A,	BV-2 Cells (LSP)	Neuroinflammation	pro-inflammatory Cytokines: NO ↓ (MAPK and TLR-4/NF-κB Pathways)	2021 [106]
Anti-oxidative effects	Fermented GLE	PC12 cells (H_2_O_2_)	Oxidative stress	Oxidative stress-related factor: LDH ↓ apoptosis-related protein: caspase-3 ↓	2015 [109]
Anti-oxidative effects	GLP	primary dopaminergic cell (MPP^+^/Rot)	PD	Free radical scavenging ability ↑ mitochondrial complex I, ΔΨm ↑ Oxidative stress-related factors: ROS ↓	2016 [110]
Anti-oxidative effects	GLP	SH-SY5Y Cells (H_2_O_2_)	Mitochondrial dysfunction	ΔΨm, SOD ↑ MDA ↓ Bax, Caspase-3 ↓ Bcl-2 ↑ fission proteins (Fis1 and p-Drp1) ↓ fusion proteins (OPA1, Mfn1, and Mfn2) ↑	2024 [111]
Anti-oxidative effects	GLT	SH-SY5Y cells (H_2_O_2_/Aβ_25–35_)	AD	Free radical scavenging ability ↑	2019 [79]
Anti-oxidative effects	GLT	APP/PS1 Transgenic Mice. (Aβ_25–35_)	AD	SOD, Nrf2, NQO1, and HO1 ↑ MDA, LDH ↓ Bax, caspase 3/cleaved caspase 3 ↓ Bcl-2 ↑ (ROCK Signal Pathway)	2020 [91]
Anti-oxidative effects	GLT	Rats (D-galactose)	Cognitive impairment	Oxidative stress-related factor: MDA, AGEs ↓ Antioxidative factor: T-AOC, GSH-Px, T-SOD, CAT ↑	2020 [102]
Anti-oxidative effects	GLS	rat hippocampus (STZ)	AD	Oxidative stress-related factor: MDA ↓ Antioxidative factor: GR, GSH, ATP and CytOx ↑	2012 [112]
Anti-oxidative effects	GLFE	PC-12 cells (H_2_O_2_)	oxidative stress	SOD, CAT, and GSH-Px ↑ PI3K and Akt ↑ Caspase-3 ↓	2024 [113]
Regulation of autophagy	GLE	Mice/Mice and neuroblastoma neuro-2a cells (MPTP/MPP^+^)	PD	protect dopaminergic neurons: TH, PINK1, Parkin ↑ autophagy related factors: BNIP3L ↑ cytochrome C, LC3-II/LC3-I ratio, AMPK, mTOR, ULK1 ↓ (AMPK/mTOR and PINK1/Parkin signaling pathway.)	2018 [11]
Regulation of autophagy	GAA	BV2 microglial cells/Rats (Aβ_42_)	AD	autophagy related factors: LC3B-II, Axl and Pak1 phosphorylation ↑ (Axl/Pak1 signaling pathway)	2021 [119]
Regulation of autophagy	GAA	HT22 cells (Aβ_25–35_)	AD	senescence and autophagy-related factors: P16, P21, Hmgal, LC3B I/II ↓ ATG5, Beclin 1, PADI4 ↑ (Akt/mTOR pathway)	2021 [97]
Stimulation of Neurotrophic factor synthesis and neurite outgrowth	GLE	PC12 cells	AD	Phosphorylation of ERK1/2 and CREB ↑	2000 [129]
Stimulation of Neurotrophic factor synthesis and neurite outgrowth	GLE	PC12 cells	AD	neurite outgrowth ↑ (MEK/ERK1/2 and P13K/Akt signaling pathways) ↑	2013 [130]
Stimulation of Neurotrophic factor synthesis and neurite outgrowth	GLP	Primary dopaminergic cell cultures prepared (MPP^+^/Rot)	PD	neurites of dopaminergic neurons ↑	2016 [110]
Promote neural stem cell proliferation	GLP	APP/PS1 transgenic AD mice	AD	NPC ↑ (FGFR and ERK/AKT pathways)	2017 [132]
Stimulate β adrenergic receptors	GAA	SH-SY5Y/ PC12 cell (SNP)	NO stress injury	adrenaline ↑ NO ↓	2020 [134]
Inhibition of AChE	GLT	Assay of AChE activity	_	AChE ↓ IC50 value = 9.40 µM~31.03 µM	2011 [136]
Inhibition of AChE	11β-hydroxy-3,7-dioxo-5α-lanosta-8,24(E)-dien-26-oic acid	Assay of AChE activity	_	AChE ↓ IC50 value = 10.8 μM	2017 [137]
Inhibition of AChE	dayaolingzhiols D and E	Assay of AChE activity	_	AChE ↓ IC50 values: Dayaolingzhiols D = 8.52 ± 1.90 μM Dayaolingzhiols E = 7.37 ± 0.52 μM	2019 [138]
Inhibition of AChE	GLBR	Assay of AChE activity	_	AChE ↓ IC50 value = 1.01 mg/mL	2013 [138]

## 5. Conclusions

The fruiting bodies of *Ganoderma lucidum* have been used to invigorate mental activity in the human brain for a long time [140]. Modern pharmacological studies suggested that *Ganoderma lucidum* could be applied to treat multiple neurodegenerative diseases through various neuroprotective mechanisms. These include anti-AChE activity, neurite outgrowth stimulation, reducing the neurotoxicity of beta-amyloid plaques, and alleviating mitochondrial dysfunction and endoplasmic reticulum (ER) stress, in addition to antioxidant and anti-inflammatory effects [62]. The interplay between the natural products from *Ganoderma lucidum* and the pathological features of NDDs suggests a multifaceted mechanism of action, offering a holistic approach to disease management that is currently lacking in conventional therapies.

Despite the advantages of lower toxicity, fewer side effects, multi-target modes of action, and potential synergistic effects with conventional treatments, the application of *Ganoderma lucidum* against NDDs may encounter several challenges and limitations during the process of drug development. Many studies on *Ganoderma lucidum* have not used pure constituents, making it difficult to attribute the observed effects to specific components of the mushroom. This limitation hampers the ability to understand the precise mechanisms of action and to develop standardized treatments. Moreover, the concentration and purity of the active compounds in *Ganoderma lucidum* can vary significantly in different preparations. This variability can affect the reproducibility and comparability of study results. In addition, more in-depth research is needed to determine the exact mechanisms and the structure–activity relationships involved in their therapeutic effects. This is crucial for understanding how these compounds can be effectively used in the treatment of neurodegenerative diseases and for guiding the modification of the chemical structure and the optimization of the formulation. Last but not least, there are a lack of comprehensive clinical trials evaluating the effects of *Ganoderma lucidum* on neurodegenerative diseases. Most studies are preclinical or observational and there is a need for more rigorous clinical research to confirm the efficacy and safety of *Ganoderma lucidum* in human subjects. A single case study involving self-medication with *Ganoderma lucidum* to combat Parkinson’s disease symptoms showed small positive changes in some facets of affective behavior. While this does not allow for firm conclusions, it suggests that clinical studies in this patient population may be warranted [141]. A randomized, double-blind, and placebo-controlled study of GLP in neurasthenia indicated that the extract was significantly superior to placebo in the clinical improvement of symptoms [142].

Given the potential neuroprotective effects, *Ganoderma lucidum* could be considered as a complementary therapy alongside conventional treatments. Its use in reducing symptoms or potentially slowing disease progression in conditions like Parkinson’s disease could be explored further. While more research is needed to fully understand and validate these effects in clinical settings, *Ganoderma lucidum* represents a novel avenue in the quest for effective and comprehensive treatment strategies for NDDs. The exploration of *Ganoderma lucidum’*s bioactivity in the context of neurodegenerative diseases not only expands our therapeutic arsenal but also provides valuable insights into the intricate mechanisms underlying these debilitating disorders. As we continue to unravel the complexities of NDDs, integrating traditional knowledge with modern scientific inquiry could pave the way for groundbreaking advancements in neurodegenerative disease treatment.

## Data Availability

No new data were created or analyzed in this study. Data sharing is not applicable to this article.
